# Subarachnoid Intra-cranial Lipiodol Deposits

**DOI:** 10.7759/cureus.63265

**Published:** 2024-06-27

**Authors:** Tom Saliba, Sanjiva Pather, Olivier Cappeliez

**Affiliations:** 1 Radiology, Hôpital de Braine-L'Alleud, Braine-L'Alleud, BEL

**Keywords:** subarachnoid, contrast media, myelography, ethiodized oil, lipiodol

## Abstract

Lipiodol, an oil-based contrast medium first introduced in 1944, was commonly used for various radiological exams until the 1980s, when it was replaced by water-soluble contrast media due to complications such as arachnoiditis and chronic irritations. Due to its slow resorption rate, asymptomatic lipiodol deposits can occasionally be found incidentally. This case report describes a 93-year-old man who presented to the emergency department after a fall. A non-contrast head CT scan, performed to rule out subarachnoid hemorrhage, revealed numerous hyperdense droplets in the subarachnoid spaces of the brain, primarily around the temporal lobes. Further investigation uncovered a previous pelvic X-ray showing similar hyperdense droplets around the cauda equina. The patient's history indicated a lipiodol myelography performed 51 years earlier. Lipiodol deposits are generally found in the lumbar region, making an intra-cranial location particularly rare. When present, these deposits are visible as radiopaque droplets on X-rays, hyperdense droplets on CT scans, and hyper-T1 on MRI, though the T2 signal is variable. Though lipiodol deposits are generally left untreated, symptomatic spinal deposits may be surgically removed. This rare case underscores the importance of thorough patient history in diagnosing subarachnoid lipiodol deposits, a condition relevant only in older patients who underwent myelography before the 1980s.

## Introduction

Lipiodol, also known as ethiodized oil, pantopaque, and myodil, is an oil-based contrast medium first used by Ramsey in 1944 [1]. It was injected via a lumbar puncture and used to perform a variety of exams, such as myelography, cisternography, and ventriculography [2]. It remained in common usage until the 1980s, after which it was superseded by water-soluble contrast mediums owing to its predisposition for causing complications, which included arachnoiditis, cysts, nerve damage, chronic irritations and arachnoid adhesions [2,3]. It is, however, possible that the lipiodol depots remain asymptomatic and are thus incidentally found during workups for unrelated pathologies [4].

Although rare, lipiodol deposits can occasionally be seen in the spinal cord or, even less commonly, the intra-cranial region [2-6].

We present the case of a 93-year-old man in whom hyperdense droplets were discovered in the subarachnoid spaces and who, after further investigation, was shown to have a history of lipiodol myelography.

## Case presentation

A 93-year-old male presented to the emergency department following a fall. The emergency department requested a non-contrast head CT scan to exclude subarachnoid hemorrhage.

**Figure 1:**
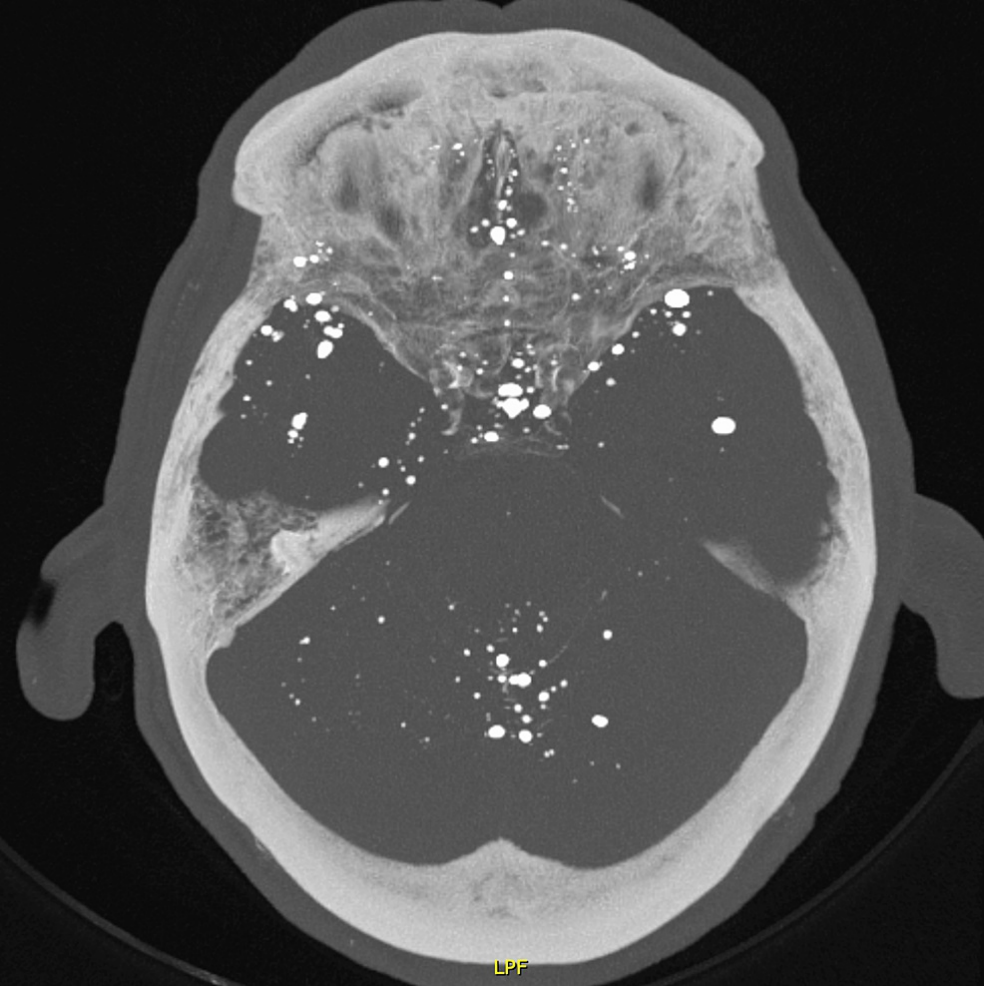
Maximum intensity projection (MIP) of an axial CT scan of the skull. MIP of an axial CT-scan image showing innumerable hyperdense droplets, situated in the temporal and frontal areas as well as within the spaces between the folia of the cerebellum.

Although the patient had no signs of acute intra-cranial pathologies, there were innumerable small hyperdense droplets of difference sizes (Figure 1). 

**Figure 2:**
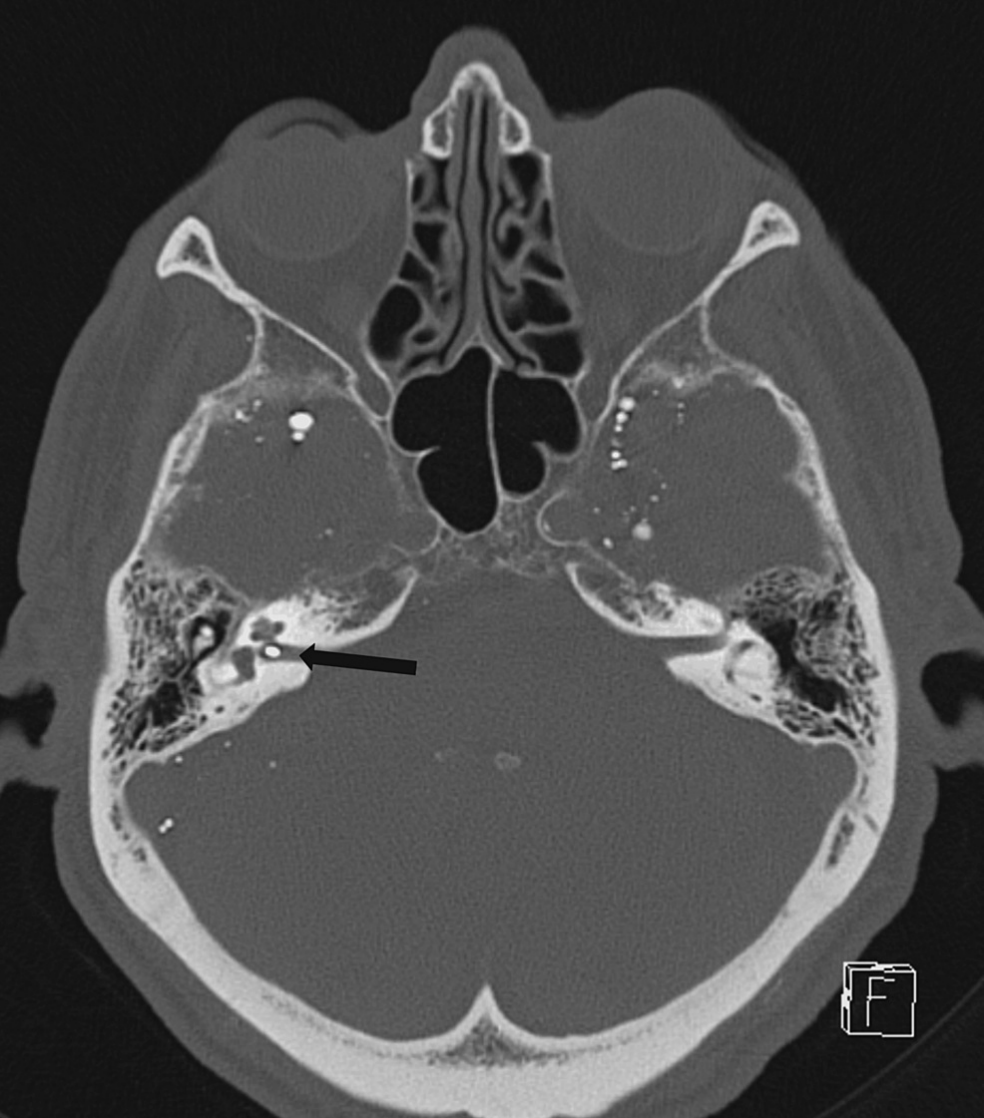
Axial non-contrast CT image of the skull. Axial non-contrast CT image of the skull showing a hyperdense droplet within the right internal auditory canal (arrow), showing that hyperdense to the adjacent petrous bone.

**Figure 3:**
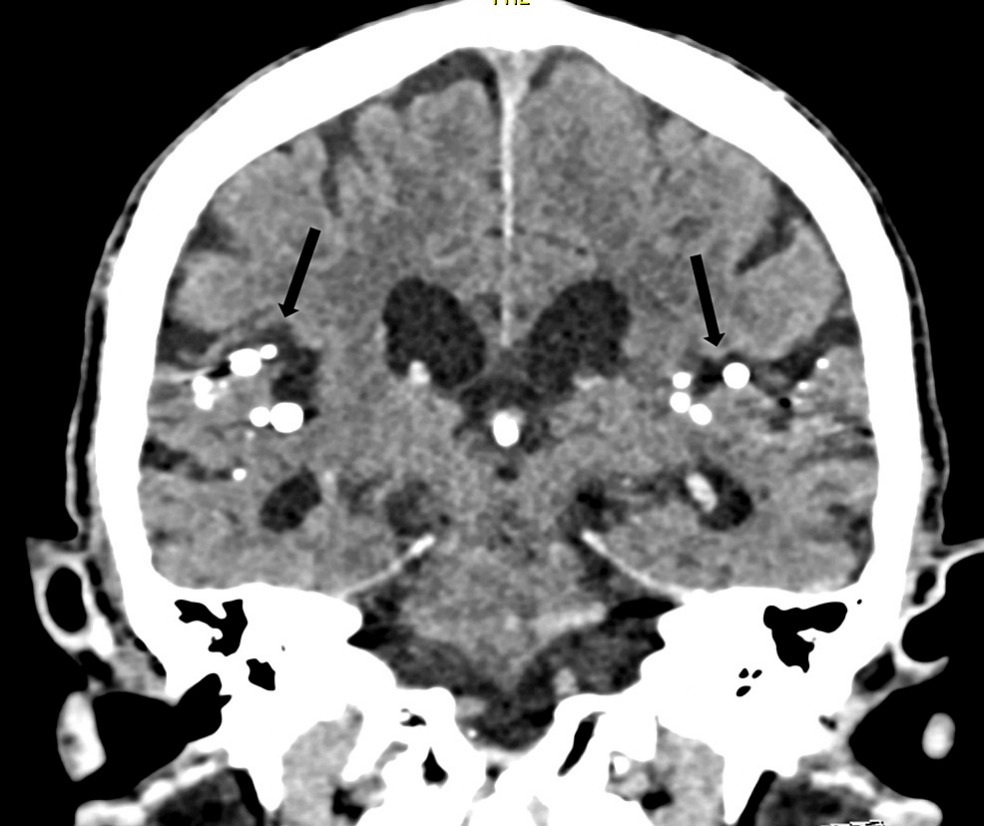
Coronal CT scan image of the skull. Coronal CT scan image of the skull showing multiple hyperdense droplets in the subarachnoid space of the Sylvian fissures (arrows).

These droplets were far denser than the surrounding bone (Figure 2) and were attached to the subarachnoid membrane surface (Figure 3), with a significant number located around the temporal lobes.

**Figure 4:**
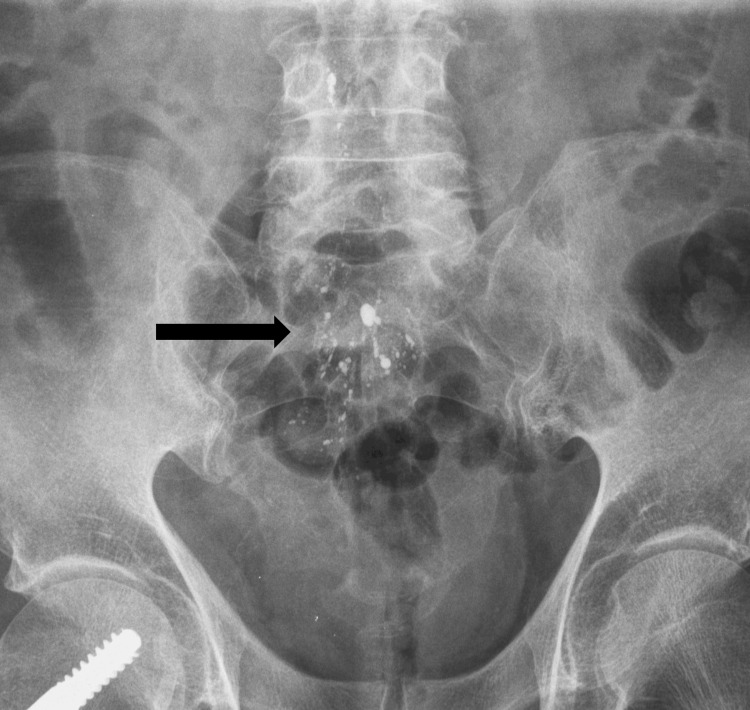
Antero-posterior lumbar X-ray. Antero-posterior lumbar X-ray showing multiple opacities around the cauda equina (arrow).

A search of the patient’s history revealed a previous pelvic X-ray which revealed multiple hyperdense droplets outlining the cauda equina (Figure 4).

The patient’s medical history revealed that he had undergone myelography using lipiodol 51 years prior. The patient was returned to the emergency room and subsequently discharged home.

## Discussion

Myelography using lipiodol, also known as ethiodized oil, can result in contrast medium deposits, though cases of intracranial deposits are exceedingly rare fewer than ten previous reports, with most other cases involving deposits in the thoracic and lumbar spine, though rare cases of thoracic deposits do exist [2-6]. As myelographies have been relegated to history, a few patients who have undergone the study still survive. 

As lipiodol is absorbed very slowly, with its half-life being reported as several months to years, it can sometimes be found in the thecal sac many years after the initial procedure [5].

Spinal remnants of lipiodol can cause arachnoiditis, spinal cord compression, back pain, paresthesia, and weakness [6]. Intracranial remnants may cause headache, arachnoiditis, hydrocephaly, meningitis, lack of balance, and vertigo, though symptoms may take up to 40 years to appear and have a gradual onset [6].

When seen on X-rays the deposits will present as small radiopaque droplets in the spine or skull [6]. A CT scan will show hyperdense subarachnoid droplets in the affected areas [6]. An MRI will show deposits that are spontaneously hyperintense in T1-weighted imaging and iso- to hyperintense in T2-weighted imaging, though a hypo-T2 appearance has also been described, and can thus mimic fat or blood [3,5]. When imaged using an MRI, the differential diagnosis includes lipomas, hemangiomas, hemorrhage, or melanomas and it is therefore useful to perform a radiograph or CT scan to prove that objects seen correspond to hyperdense lipiodol deposits [5].

The removal of intracranial deposits has been reported, though no guidelines or clear indications for doing so exist [6]. Spinal deposits may be removed if they are symptomatic [6].

## Conclusions

In conclusion, we presented the case of a 93-year-old male in whom hyperdense droplets were discovered during a head CT performed as part of a post-traumatic workup. Through a careful history, it was revealed that the patient had undergone myelography with lipiodol, 51 years prior to the current presentation, confirming the diagnosis of subarachnoid lipiodol deposits. In suspected cases of lipiodol deposits, a thorough patient history is essential to establish a definitive diagnosis. This entity should only be suspected in patients old enough to have undergone myelography, a practice that ended in the 1980s.
